# Servelle-Martorell syndrome in an adult: A case report with findings on CT angiography

**DOI:** 10.1016/j.radcr.2021.03.023

**Published:** 2021-04-10

**Authors:** Anh Tuan Tran, Cuong Tran, Nguyen Quyen Le, Thi Huyen Nguyen

**Affiliations:** a Radiology Center, Bach Mai Hospital, Ha Noi, Vietnam; b Department of Thoracic, Vascular and Neurology, Trung Vuong Hospital, 266 Ly Thuong Kiet, District 10, Ho Chi Minh city, Vietnam

**Keywords:** Servelle-Martorell syndrome, Venous malformations

## Abstract

Servelle-Martorell syndrome, also known as angio-osteohypotrophic syndrome, is a congenital venous malformation that rarely involves the arterial system. There are many different, mainly conservative, treatment methods. We present the case of a 28-year-old woman with a history of left arm and back lesions, which appeared at birth and had enlarged over time. She had been diagnosed with a vascular malformation but had not yet received treatment. She presented to our hospital with numbness in her left arm and was diagnosed with Servelle-Martorell syndrome based on computed tomography (CT) angiography. This case emphasizes that CT angiography is a useful modality for evaluating the extent of the venous malformations in patients with Servelle-Martorell syndrome.

## Introduction

Servelle-Martorell syndrome, also known as Servelle-Martorell angiodysplasia, is a rare congenital venous malformation seldom involving the arterial system [1,2]. This syndrome is characterised by sinuous dilatation of superficial veins and bony hypoplasia, causing limb hypertrophy, usually of the upper limb; therefore, it is also known as angiodysplasia-osteohypotrophic syndrome. There are many different treatment methods, including conservative ones [3]. Servelle-Martorell syndrome has rarely been reported in the literature. There are no studies with a sample size large enough to assess the prognosis [1], [2], [3], [4].

## Case presentation

A 28-year-old woman presented to our hospital with numbness in her left arm. Her left arm and back had been larger than the contralateral side since birth and this had increased over time with partial functional difficulty. Sometimes, she felt numbness in the left arm, which worsened when it was lowered. She had been examined at various hospitals and diagnosed with a vascular malformation but had not yet received any treatment.

The patient had experienced severe numbness in her left arm for the previous two weeks. This was not relieved by painkillers. It was often necessary to lift the arm above her head in order to feel more comfortable.

**Fig. 1:**
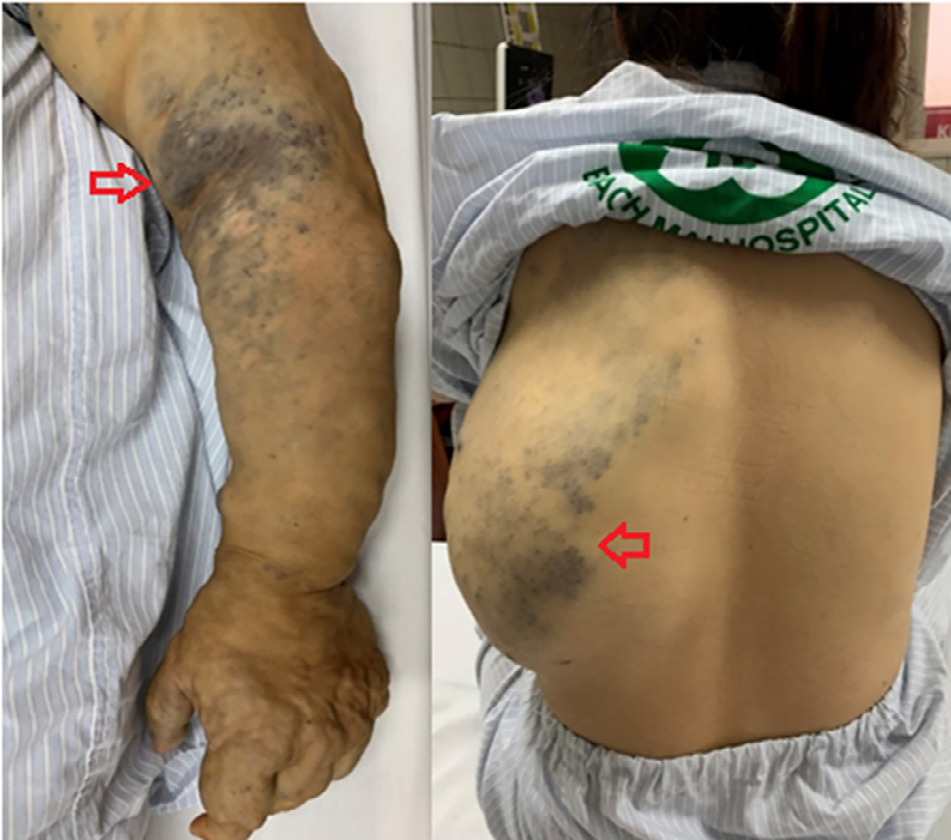
The left arm and back were large and deformed with bluish tint (red arrows). (Color version of figure is available online.)

On physical examination, the entire arm and part of the left back were deformed, being larger than the opposite side (Fig. 1), but the two arms were of equal length. The skin on the left elbow, hand, and back had a bluish tint. The affected region had multiple swollen areas which were different in size, squash, and easily compressible. Some hard round particles could be felt under the skin. The peripheral pulses were symmetrically palpable, and no temperature difference was observed. No abnormalities were found in the cardiovascular system or in other organs.

Blood tests demonstrated higher than normal levels of D-dimer (> 7.65 mg/L compared to the reference value of < 0.48 mg/L), and the prothrombin time (PT), prothrombin time ratios (PTr), International normalised ratio (INR) values and platelet count were within normal ranges.

An ultrasound examination of the left arm revealed numerous subcutaneous tubular structures. These were thin-walled with an anechoic lumen, completely compressible, and colour flow was present with low velocities on Doppler ultrasound. These features were consistent with the telangiectatic superficial veins.

**Fig. 2:**
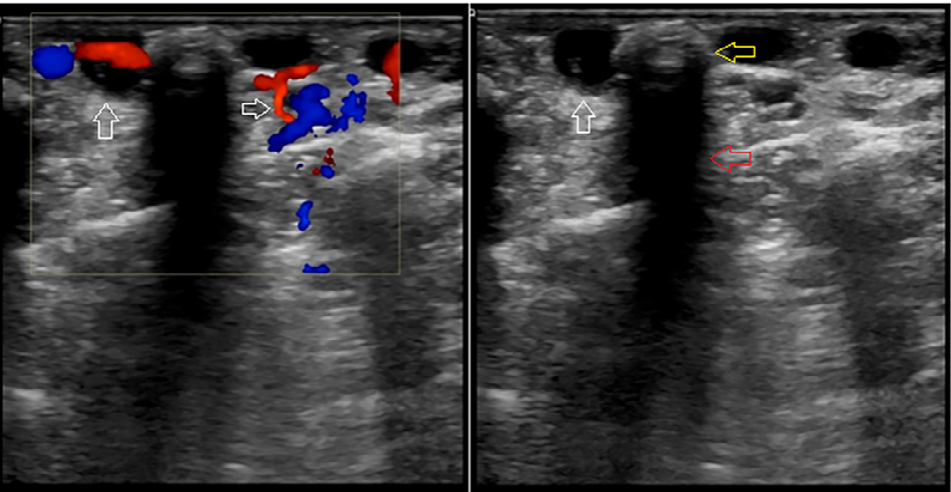
The anechoic, completely compressible structures just below the skin surface had a colour signal on the Doppler ultrasound suggestive of telangiectatic superficial veins (white arrows). Inside these structures were phleboliths (yellow arrow), appearing as echogenic particles with posterior acoustic shadowing (red arrow). (Color version of figure is available online.)

Inside these structures were many echogenic particles with posterior acoustic shadowing consistent with phleboliths (Fig. 2). The axillary, brachial, ulnar, and radial arteries had normal diameters, velocities, and waveforms. The accompanying deep veins did not dilate, were completely compressed, and no thrombosis was observed.

**Fig. 3:**
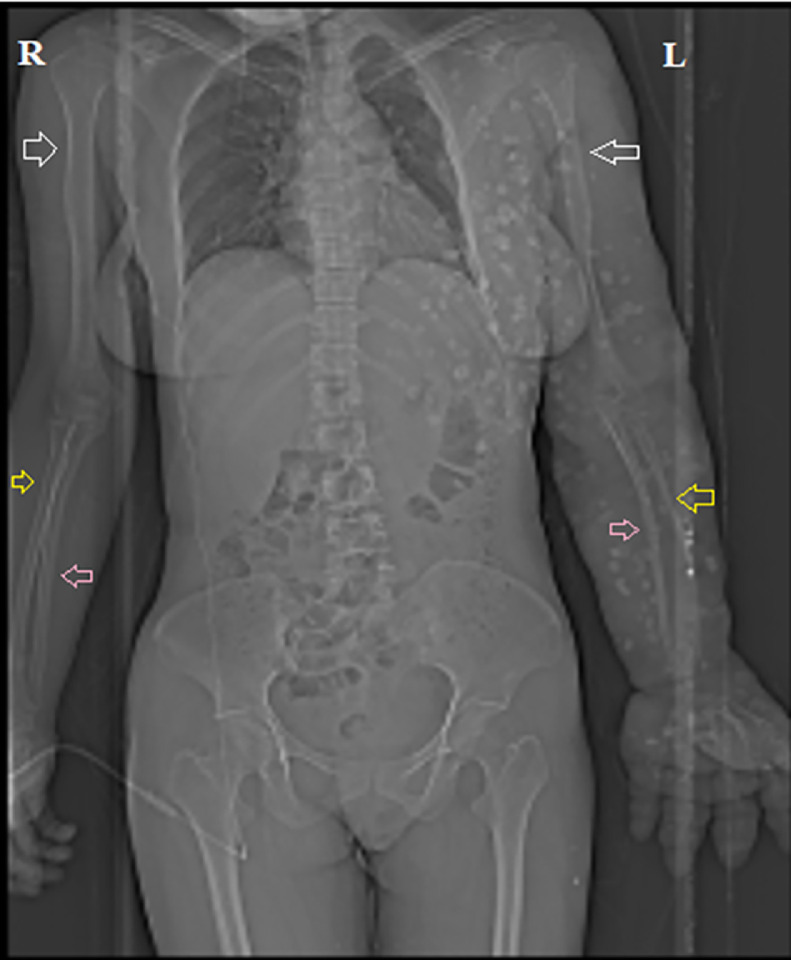
Topogram showing left (L) humerus (white arrows), ulna (pink arrows) and radius (yellow arrows) smaller than the opposite side (R). (Color version of figure is available online.)

The topogram film showed that the humerus, ulna, and radius on the left side were smaller than those on the right side. Multiple well-defined radiopaque lesions were distributed along the left upper limb, chest, and back (Fig. 3).

**Fig. 4:**
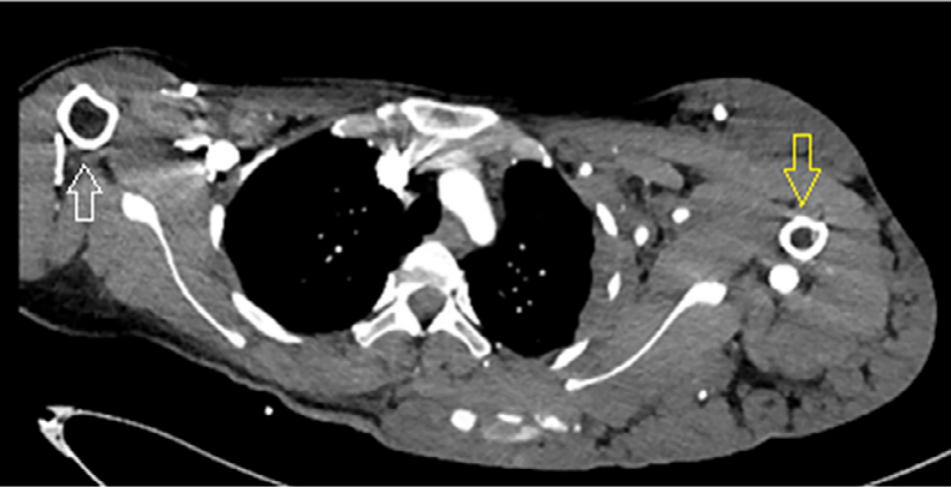
Computed tomography showing the diameter of the left humerus (white arrow) smaller than the right (yellow arrow). (Color version of figure is available online.)

**Fig. 5:**
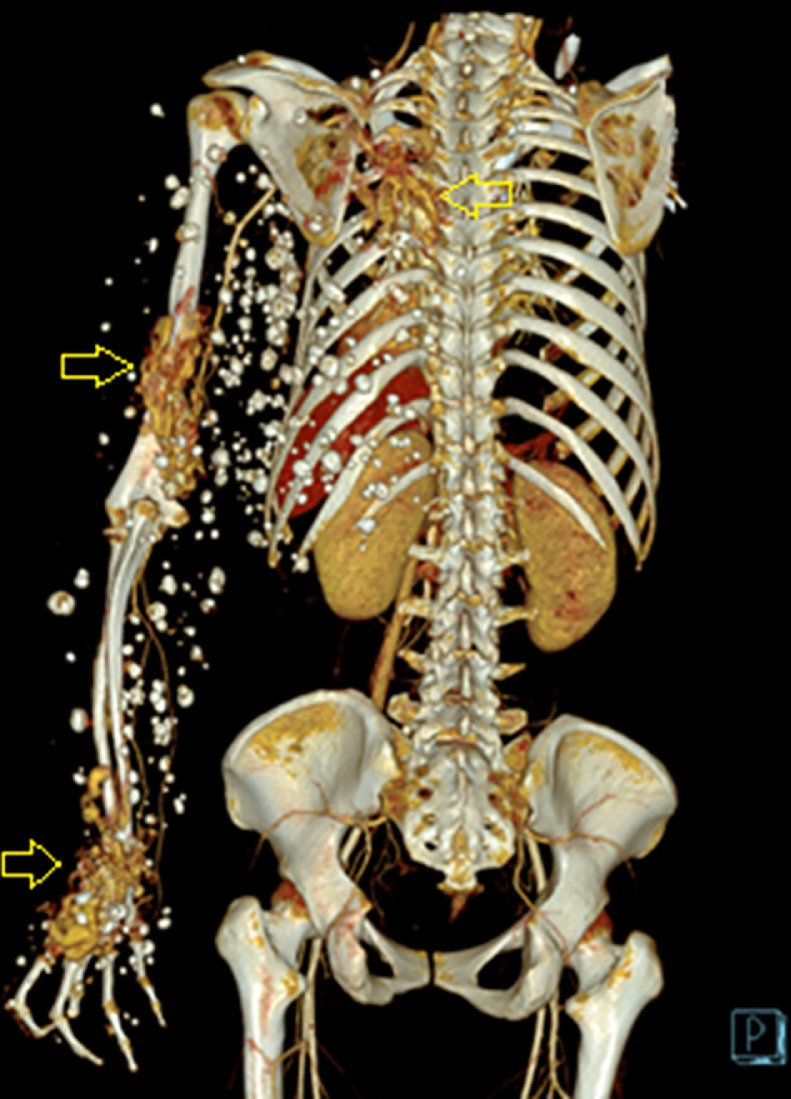
Three-dimensional image demonstrating arteriovenous fistulas on the left back, posterior aspects of arm and hand (3 yellow arrows) with many calcified particles distributed along the left arm, chest, and back. (Color version of figure is available online.)

**Fig. 6:**
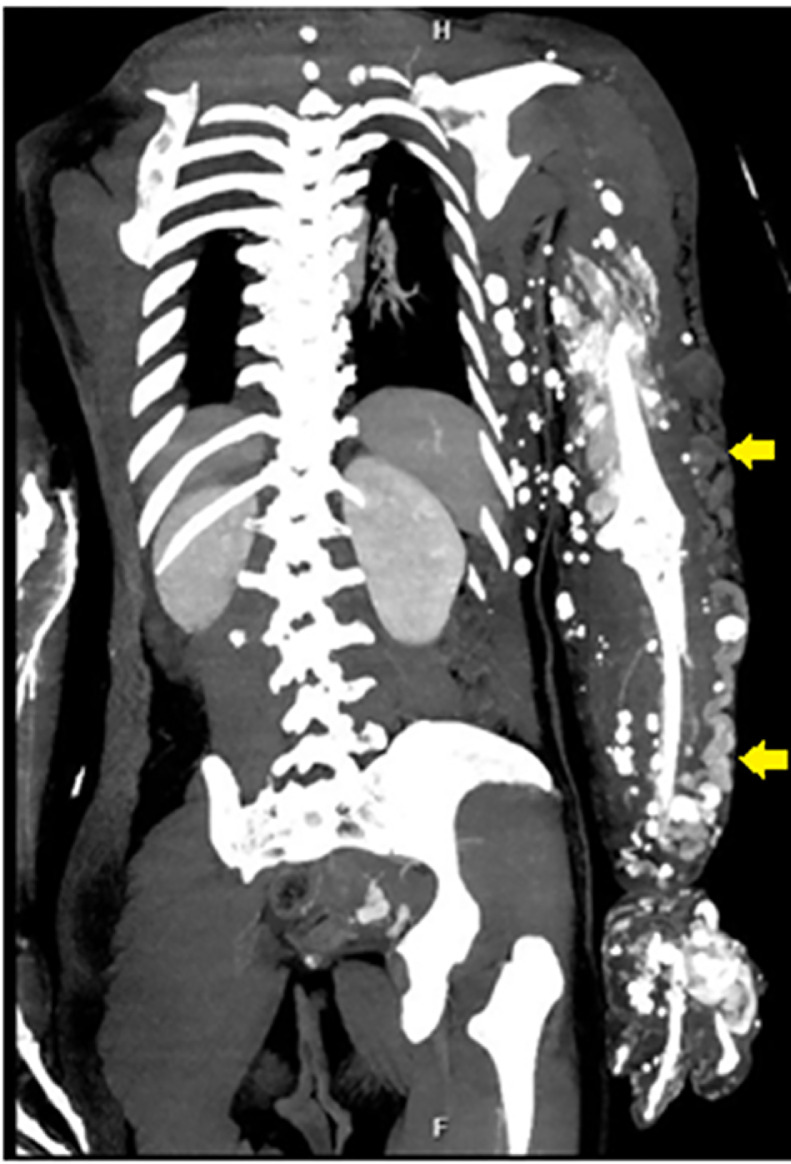
The telangiectatic superficial veins (yellow arrows) as seen in the venous phase of computed tomography scan. (Color version of figure is available online.)

A computed tomography scan of the left arm also revealed that the diameter of the left humerus was smaller than that of the right (Fig. 4). The arterial phase showed the axillary, brachial, ulnar, and radial arteries with normal courses and early enhancement of some veins. In addition, there were three vessel plexuses on the left back and posterior aspects of the arm and hand, with enlarged feeding arteries originating from the left third intercostal artery, brachial artery, common palmar digital arteries, with suspected arteriovenous fistulas. In the venous phase, scattered calcified round or oval particles were observed within dilated sinuous superficial veins (Figs. 5 and 6).

The patient was diagnosed with Servelle-Martorell syndrome and treated with conservative methods, including wearing compression stockings and taking Daflon. However, the blood in the left arm was evacuated to the shoulder when she used a compression sock, making sleep difficult, so she stopped wearing it, and kept her left hand held high to decrease the numbness.

## Discussion

Servelle-Martorell syndrome is a rare congenital venous malformation with diverse lesions. The superficial veins are sinuous and dilated, resulting in deformity in the affected limb, phlebothrombosis, and phleboliths. The deep veins may be anaplastic and have abnormal courses associated with the partial or complete absence of valves. Vascular malformations in the bones lead to destruction of the spongy bone and bony cortex, resulting in shortening of the limbs [5]. In the case of the patient described, the left upper limb and the back were distorted by vascular malformations. The humerus, radius, and ulna were hypoplastic, though there was no limb shortening due to limb hypertrophy secondary to telangiectatic superficial veins. There were many scattered phleboliths located within the anomalous vessels.

Most of the cases reported in the literature involve a part of the limb, but not the entire upper limb as in this clinical case.

A study by Langer et al., conducted on 47 patients, found that a diagnosis of Servelle-Martorell syndrome can be confused with some similar complex-combined disorders, such as Parkes-Weber syndrome and Klippel-Trenaunay syndrome. Venous malformation is found in all these syndromes, while bony hypoplasia in association with limb hypertrophy is characteristic of Servelle-Martorell syndrome [6].

The entire left arm and part of the left back were enlarged and deformed, resulting in problems for the patient. It not only caused feelings of numbness and functional difficulties, such as in holding objects and wearing clothes, but also caused embarrassment due to her abnormal appearance. She always desired to be cured and resemble other people.

There are many different treatment methods, including external compression, sclerotherapy, and surgery; the majority of cases are treated conservatively [1]. Compression stockings are useful in protecting the limbs from injuries that may cause bleeding from the dilated veins just below the surface of the skin. However, they do not minimize the bulk. It was suggested that the patient use a compression stocking on the left arm; however, the blood flowed to the back, which caused her discomfort, so she stopped using it. The patient's symptoms were not relieved. She regularly put her left hand above her head to decrease the numbness.

Sclerotherapy is indicated for small lesions. Surgical resection of varicosities can be achieved after successful sclerotherapeutic obliteration.

Surgery is rarely performed unless there are complications, such as aneurysms or severe shunts. Establishing decisions for surgical intervention must be based on multiple consultations and careful preparation [3]. In some cases, excision of localized telangiectatic veins can be effective if the deep vein system has good function.

In addition, diuretics are used in patients with significant swelling of the limbs. Prophylactic antibiotics should be used in cases of recurrent vasculitis. Anticoagulants are indicated in the presence of deep vein thrombosis or pulmonary embolus. If the patient has recurrent superficial thrombosis, it is necessary to take aspirin or ibuprofen daily, but this increases the risk of bleeding increases, so care must be taken if the patient is active.

## Conclusion

Servelle-Martorell syndrome is a rare disorder characterized by venous malformations and bony hypoplasia associated with limb hypertrophy, usually in the upper extremity. There are various treatment methods, such as external compression, sclerotherapy, and surgery. Conservative management is indicated in most patients. The prognosis of this condition remains uncertain.

## Patient consent

Written informed consent was obtained from the patient for publication of this manuscript.
